# Supplementary material for: Are carbon emissions trading and green financial instruments synergistic? -Comprehensive quantitative research based on content analysis
Source: PLoS One. 2024 Mar 7;19(3):e0298601. doi: 10.1371/journal.pone.0298601 (PMC10919601; doi:10.1371/journal.pone.0298601)
Supplement: S1 Data — (ZIP) [file pone.0298601.s001.zip › Data/地级市Z_F工作报告.zip!/╣π╬≈/║╙│╪╩╨/└┤╫╘╣1⁄2╓┌║┼í╛┬φ┐╦╩2╛▌═°í┐_2019.docx]

各位代表：

　　现在，我代表市人民政府向大会报告工作，请予审议，并请市政协委员和列席会议的同志提出意见。

　　一、2018年工作回顾

　　刚刚过去的一年，我们在以习近平同志为核心的党中央坚强领导下，以习近平新时代中国特色社会主义思想为指导，全面贯彻党的十九大精神，在自治区党委、政府和市委的正确领导下，在市人大、市政协的监督支持下，认真落实“三大定位”新使命和“五个扎实”新要求，深入开展“六大行动”提质年活动，推动全市经济社会持续健康发展，较好完成了市四届人大三次会议确定的目标任务。预计全市实现地区生产总值796亿元，同比增长7%；规模以上工业总产值增长15%，增加值增长8%；固定资产投资增长15.6%；财政收入77.5亿元，增长11.7%；社会消费品零售总额332.8亿元，增长10.5%；全体居民人均可支配收入15869元，增长9.2%，其中城镇居民人均可支配收入27314元，增长6.5%，农村居民人均可支配收入9127元，增长10.5%；外贸进出口总额29亿元，增长48.4%，其中出口总额4.6亿元，增长106.7%；居民消费价格涨幅为2.5%，控制在3.5%目标任务内。全市经济社会发展呈现许多新亮点。

　　（一）脱贫攻坚取得重大进展。扎实开展打赢脱贫攻坚战三年行动，集中力量攻坚深度贫困，不断夺取了脱贫攻坚新胜利。2018年有望减贫17.27万人、146个贫困村出列、金城江和天峨脱贫摘帽，贫困发生率从上年的13.06%下降到8.19%。强力推进易地扶贫搬迁，累计搬迁入住16.62万人，为全区最多，已完成“十三五”搬迁任务的90%。贫困地区基础设施大幅改善，新建通屯道路900公里，已累计建成2469公里，基本完成“十三五”目标，所有具备条件的20户以上自然屯通路；巩固提升33.8万群众饮水安全；实施危房改造1.6万户；解决2.4万户用电难问题；行政村通宽带率100%。公共服务水平不断提升，22.4万贫困劳动力实现转移就业，35.8万贫困学生得到有效资助，劝返辍学学生2597人，贫困群众住院报销比例提高到90%以上，农村低保标准提高到每人每年3800元，超过国家年度扶贫标准。生态扶贫精准发力，兑现森林生态效益补偿、退耕还林补助3.7亿元，新增护林员补助7500万元，带动3.84万人脱贫。在为69.1万贫困群众、贫困村第一书记购买意外伤害险的基础上，又为市县4.58万名帮扶干部投保。新增中国人保财险公司2亿元产业低息贷款，“政府平台+融资保险”产业发展机制在全国推广。95%的贫困村集体经济收入从无到有，508个年收入超过3万元，全区发展壮大农村集体经济工作经验交流会在我市召开。脱贫攻坚资金投入持续加大，“十三五”以来累计投入452亿元，其中2018年筹措财政专项资金、整合涉农资金超100亿元，同时强化资金监管，确保扶贫资金用到惠民实处。深圳·河池扶贫协作持续深化，落实帮扶资金2.4亿；7个国家定点帮扶单位和区直有关部门帮扶成效明显，直接投入资金1.8亿元，全国携手奔小康现场会在我市召开。深入开展“千企助百村”精准扶贫行动，363家民营企业结对帮扶519个贫困村，投入资金物资9.6亿元，统一战线助力脱贫攻坚经验在全区推广。全市政协组织开展“助一村、帮一户、献一策”助力精准脱贫活动，捐资捐物6785万元，帮助协调引进产业项目486项，实施基础设施项目1650项，成为全区政协“乡村振兴委员行，助推脱贫攻坚”工作的典范。

　　（二）“十大百万”扶贫产业工程和“三农”工作成效显著。把产业发展作为乡村振兴和脱贫攻坚的主抓手，基本实现扶贫产业对贫困户的全覆盖，有效提高了脱贫质量，带动第一产业增加值增长6%，比上年提高2.1个百分点。全区先后4次在我市召开产业扶贫现场会，典型经验和做法得到央视、新华社等主流媒体多次聚焦报道。全市建成核桃“万千百”示范基地34万亩，被列为国家森林生态标志产品（核桃）生产基地创建试点市；桑园总面积90万亩，连续14年居全国第一；油茶总面积125万亩，建设油茶千亩高产示范基地24个，列为全国木本油料特色区域示范市；创建7个种牛、10个种羊繁育示范基地，出栏肉牛26万头、肉羊107万只，肉牛、肉羊饲养量及出栏量居全区第一；“三特”水果、糖料蔗、板栗、香猪、淡水养殖、长寿生态富硒农产品等产业完成年度目标任务。都安“贷牛还牛”全产业链发展模式成为全区扶贫产业的样板，都安肉牛养殖现代特色农业示范区成为我市首个，也是全区唯一一个以肉牛为主导产业的自治区五星级示范区。罗城高产油茶种植，东兰油茶低产林改造，天峨“三特”水果，凤山核桃管护，成为全市示范，宜州区被农业农村部评为全国农村创业创新典型县。

　　在“十大百万”扶贫产业的引领下，乡村振兴战略实现良好开局，农业农村工作迈上了新台阶。粮食生产保持安全稳定。全市累计创建各类示范区1122个，经营总收入17.3亿元，直接带动18.5万农户增收，新认定11个自治区级现代特色农业示范区，总量达25个，创建数量和认定数量排全区前列。新建现代特色林业示范区183个，林下经济示范基地14个，林下经济产值57亿元。农田水利基础设施持续加强，新增、恢复和改善灌溉面积13万亩。耕地保护全面夯实，连续19年实现耕地占补平衡。完成天峨特色水果和宜州桑蚕生产机械化示范项目建设，农作物耕种收综合机械化率达52.5%，荣获“全国平安农机示范市”，是全国表彰的10个示范市之一，也是广西唯一获得的示范市，天峨、罗城荣获“全国平安农机示范县”称号。大力实施农村人居环境整治三年行动，持续推进“美丽河池·宜居乡村”建设，累计建成32个乡镇污水处理厂，所有行政村建成综合服务中心，完成13.5万户改厕、13.5万户改厨、3.5万户改圈任务，群众生产生活条件明显改善。

　　（三）工业经济保持良好发展势头。继续加大工业转型升级力度，工业总量不断壮大，有色金属、茧丝绸、农产品加工、建材等特色优势产业持续加快发展，产值分别增长18%、16%、20%、60%。园区建设步伐加快，市级工业园区完成总产值183亿元、增加值35亿元，引进亿元以上项目10个。大任产业园累计完成投资56亿元，实现“五通一平”目标，已有12家企业进驻，5家企业投产，完成产值7亿元。加大龙头企业扶持力度，一批重点企业加快发展，南方公司实现产值130亿元、销售收入150亿元。工业发展后劲不断增强，超威鑫锋公司新型蓄电池（一期）、华远公司年产1.5万吨锑品深加工、生富公司年产1.5万吨锑金属技改、琦泉生物质发电等一批项目竣工投产；南方公司3万吨锡冶炼、誉升公司10万吨锌等一批新项目开工建设。优质饮用水、酿酒产业不断壮大，产值分别增长30%、9%。浪潮河池云计算中心建设加快推进，完成政务云平台搭建、机房和创客中心建设。绿色制造体系建设取得新进展，南方公司等3家企业成功入选国家级绿色工厂，丽琅公司等2家企业入选自治区绿色工厂。培育高新技术企业10家，南方、华远2家企业列为自治区首批创新驱动发展重点扶持企业。主动做好服务企业工作，帮助企业降低用电成本2亿多元，协调企业融资18亿元，新培育规模以上工业企业25家。质量强市战略扎实推进，5个产品荣获“广西名牌产品”称号，成功申报地理标志商标13个，数量排全区第一，茧丝品牌和质量备受市场青睐，全国茧丝质量技术比对交流活动在我市举行。

　　（四）以全域旅游为重点的现代服务业蓬勃发展。全市接待游客增长26.7%，旅游总消费增长35.7%。巴马国际旅游区、红水河健康养生之旅、百里龙江旅游带的重点项目加快推进，新建成巴马洞天福地、南丹丹炉山、罗城棉花天坑等景区，新增4A级景区5个，新（改）建“旅游厕所”103座，建设11个三星级以上汽车旅游营地。“世界长寿市”品牌更具影响力，巴马、宜州列为国家全域旅游示范区创建县，环江、罗城荣获全国森林旅游示范县，所有县（区）入围自治区创建特色旅游名县、创建全域旅游示范区“双创”名单，南丹成功创建为全区全域旅游示范区。商贸物流业稳步发展，都安西南冷链仓储物流中心投入使用，红星美凯龙商业广场、都安畜禽交易中心等项目加快建设；积极开展展会、电商促销活动，拉动消费增长10亿元；外贸进出口完成29亿元，是近十年来取得的最佳成绩；罗城、环江、凤山获批全国电子商务进农村综合示范县，实现所有县份的全覆盖，示范县数量、项目资金均排全区第一。全市新增存款62亿元，存款余额1190亿元；新增贷款60亿元，贷款余额721亿元。保险创新发展，实现保费收入20.9亿元。强化政府增信，新增担保额4.4亿元，担保余额5.2亿元。

　　（五）以交通、水利基础设施为重点的项目建设实现重大突破。紧紧抓住国家新一轮扩投资补短板政策机遇，围绕基础设施和产业发展薄弱环节，谋划项目1115个，总投资5000亿元，项目数量和投资额大幅提高，针对性和有效性显著增强。集中力量推进项目建设，实施自治区级、市级层面统筹推进重大项目424项，完成投资335亿元，占固投比重70%，项目个数和投资体量再创新高。争取到2017年度中央预算内投资项目159个、16.8亿元，开工率、年度投资完成率均达100%；2018年度项目84个、10.2亿元，开工率100%。交通基础设施建设取得历史性突破，公路项目年度完成投资首次突破100亿元，在建高速公路总里程369公里，创历史最大规模，河池至百色高速公路建成通车，结束了“东巴凤”三县不通高速公路的历史，全线贯通广西大西环高速路网；贺州至巴马、融水至河池、乐业至百色（天峨段）高速公路项目建设加快推进；投资224亿元的南丹至天峨下老高速公路开工建设；新谋划9条15个路段815公里高速公路项目，列入新修编的广西高速路网规划，新增总里程仅次于南宁市，建成后可实现每个县（区）通两条以上高速公路。建成罗城至融水洞头、罗城至柳城六塘、东兰武篆至泗孟、东兰长乐至巴社山等路网项目；开工建设东兰经大化板升至都安大兴、罗城四把至环江等二级公路；抓好 “四好农村路”建设，凤山成功创建“四好农村路”自治区示范县，金城江成为达标县，全市行政村道路硬化率100%，通客车率81%。贵阳至南宁高速铁路（河池段）全面开工建设，完成投资22.5亿元，超额完成年度征地拆迁任务，得到自治区通报表扬；柳州至河池城际铁路项目前期工作推进方案获得自治区政府批复。水利基础设施建设大会战进展顺利，90个重点项目完成投资12.5亿元。建成凤山乔音水库扩容、罗城白坝、东兰香河、凤山社更4个水库，开工建设金城江水电站、巴马所略水库扩容等项目，完成土桥水库除险加固，完成桂西北扶贫治旱——红水河灌区工程规划报审。电力基础设施建设得到加强，完成电网建设投资9.6亿元，建成8座变电站，改造升级农村台区2100个，供电能力和供电可靠性显著提升。

　　（六）新型城镇化进程步伐加快。河池市城市总体规划修编获批实施，金城江城区控制性详规修编成果即将批复，高铁新区、宜州城区、宜州新区（一期）以及各县城区控制性详规编制工作稳步推进。市行政中心迁移工作有序开展。自治区政府原则同意将金宜一级公路无偿移交我市。抓好中心城区重点项目建设，金城江城区的市工人文化宫、五小碧桂园校区、道路“白改黑”（一期）、城乡生活垃圾焚烧发电等一批项目竣工，市体育场、辉乐桥、乾宵路改造、路灯节能改造（黑改白）等一批项目加快推进；宜州城区的浙江大学西迁宜山纪念馆、河池·宜州政协文史馆、宜州刘三姐文化博物馆、西屏实验学校等项目投入使用，环城路、“九街十八巷”改造等项目稳步实施；大力推进“智慧城市”建设，“城市管家”服务平台投入运行。城市综合管理水平进一步提升，拆除“两违”建筑6.22万平方米。大县城战略深入实施，统筹推进县城基础设施项目75项，建成一批各具特色的县城和小城镇，宜州刘三姐全国特色小镇完成规划编制，巴马养生养老小镇、南丹丹泉小镇、宜州天丝小镇、东兰铜鼓小镇列为首批广西特色小镇。17个百镇示范、乡改镇、少数民族乡基础设施项目积极推进，累计完成投资2.5亿元。保障性安居工程建设进度加快，市本级的人民厂、老虎山两大片区棚户区改造项目全面推进，累计完成投资15.9亿元；全市基本建成棚户区改造5817套，累计分配入住公共租赁住房3.45万套。

　　（七）生态环境优势得到全面巩固。大力践行“绿水青山就是金山银山”发展理念，打好污染防治攻坚战，坚决守住了生态红线和环境安全底线，擦亮了“山青水秀生态美”的金字招牌。以中央环保督察反馈问题整改为契机，切实解决了一批长期难以解决的历史遗留问题，中央第六环保督察组反馈的11个问题全部完成整改，群众向“回头看”督察组反映的问题也全部办结，我市以环境倒逼机制推动产业转型升级的经验做法得到中央环保督察组充分肯定。率先在全区打响大气、水、土壤污染防治“三大战役”，加大工业废气、城市扬尘、机动车尾气治理，禁止城区燃放烟花爆竹，金城江城区环境空气优良率94.49%，空气质量改善率全区第一；全面推行“河长制”，通过自治区考评验收，总分排全区第二，红水河、龙江河、刁江等主要河流水质全面改善，100%达标，实现了河畅水清、岸绿景美；土壤污染综合防治先行区建设扎实推进，累计争取中央专项资金6.9亿元，逐步形成源头预防、风险管控的土壤治理与修复模式。开展森林和湿地综合效益评估工作，启动创建国家森林城市，“林长制”成为全区试点，完成植树造林39.7万亩，森林覆盖率69.74%，稳居全区前列。深入开展石漠化治理，治理岩溶441平方公里、治理石漠化167平方公里。投入1663万元治理地质灾害点45处。

　　（八）改革开放持续深化。全面落实“三去一降一补”，化解煤炭过剩产能72万吨，占全区任务的88.9%；持续降低实体经济成本，减免税款20.3亿元，减征社保费1.16亿元。积极抓好地方机构改革。深化“放管服”改革，推进政务服务“一网、一门、一次”工作，整合后新的“一站式”政务服务中心已完成装修，市本级政务服务事项网上办理率达91%，比自治区要求高出11个百分点；扎实推进商事制度改革，全市新增市场主体2.02万户；深入推进不动产登记制度改革，成为首个与自治区不动产数据联网并实现“最多跑一次”的设区市，得到李克强总理的肯定和批示；企业开办仅用2个工作日，是全区目前企业开办办结时间最短的设区市。积极推进国资国企改革，基本完成“三供一业”分离移交，新组建文化旅游投资公司、国林集团、金山湖投资公司；巴投公司总资产、营业收入均突破100亿元。农业农村改革迈出新步伐，基本完成农村土地承包经营权确权登记颁证工作；全面完成国有林场主体改革任务，持续深化集体林权制度改革，市本级和环江列为全国集体林业综合改革试验区。城市管理、文化教育、医疗卫生、社会治理等领域改革不断推进。宜州区合寨村的韦焕能同志作为“基层群众自治制度的探索者”，荣获“全国改革先锋人物”称号。对外开放合作力度进一步加大，与广西科学院共建河池分院，探索院地合作新机制，共享科技资源和成果转化；深圳·巴马大健康合作特别试验区提升为粤桂扶贫协作重点项目，引进深圳企业43家，合作项目58个，计划投资309亿元。所有县（区）推行招商引资项目代办服务制，为125个企业代办审批事项240项，全年招商引资内外资到位381亿元。

　　（九）社会民生得到明显改善。教育事业不断发展，新建幼儿园22所，获自治区新认定多元普惠幼儿园82所；环江、南丹、天峨、东兰、凤山、大化6县义务教育均衡发展通过国家督导评估认定，宜州、罗城、巴马3县（区）通过自治区督导评估；高中教育质量进一步提升，高考上一本录取线2445人，获清华、北大录取19人，创历史新高；广西现代职业技术学院、市职教中心的办学实力居全区同类学校前列；金城江、罗城代表广西高分通过国家通用语言文字工作督导评估。就业创业工作有序推进，全市城镇新增就业1.6万人，新增农村劳动力转移就业5.8万人次。卫生健康事业长足发展，建立医疗联合体374个，建设53个乡镇卫生院项目；市三医院晋级国家三级综合医院；环江、天峨、东兰、大化成功创建自治区卫生县城，实现所有县（区）全覆盖；“全面两孩”政策有效落实。社会保障水平不断提高，社会保险参保人数650万人次，基金征缴收入23.5亿元；落实职工医疗双保险制度，受益3055人次，二次报销医疗费1260万元；在全区率先将社会救助事项审批权限下放至乡镇，救助城乡低保对象、困难群体398万人次。文体事业全面发展，成功举办15省24市政协书画联展、滇黔桂3省10市老年人体育协作赛、第十九届河池铜鼓山歌艺术节、“壮族三月三·河池嘉年华”、市第六届运动会、第三届环红水河山地自行车赛等活动400多场次，城乡群众性文体活动丰富多彩；各类竞技体育赛事成绩喜人，荣获第十四届广西少数民族传统体育运动会金牌18枚，列金牌榜首位；罗城荣获全国体育先进县。创新矛盾纠纷多元化解机制，扎实推进信访积案化解，深入开展扫黑除恶专项斗争、禁毒严打整治等专项行动，推进全面从严治警“五查五整顿”，成功破获“8·05”特大跨国贩毒案、“1·06”御中原网络传销案、“8·30”特大“六合彩”网络赌博案等大案，“基本解决执行难”工作排在全区前列，政法机关维护稳定和服务发展的能力提升、作用凸显，实现了“六个不发生”，群众安全感达96.27%，高于全区平均水平，凤山连续三年获评全国信访工作“三无”县。全面落实优抚安置政策，推进双拥共建和国防后备力量建设。严格落实地方党政领导干部安全生产责任制，加大安全生产专项整治力度，安全生产形势持续稳定。加大土地矿产卫片执法检查，市本级连续8年实现“零约谈、零问责、零曝光”。市县同步创建食品安全城市，市本级初步通过验收。深入开展民族团结进步创建，21个集体和26名个人荣获自治区第八次民族团结进步表彰。成功举办大化瑶族自治县成立30周年县庆活动。圆满完成自治区成立60周年各项庆祝活动，热情迎接中央代表团慰问，展现了我市各民族共同团结奋斗、共同繁荣发展的良好形象。

　　（十）政府自身建设不断加强。认真学习贯彻习近平新时代中国特色社会主义思想和党的十九大精神，推进“两学一做”学习教育常态化制度化，树牢“四个意识”，增强“四个自信”，以实际行动做到“两个维护”。坚决贯彻中央，自治区党委、政府和市委的决策部署，力戒形式主义和官僚主义，狠抓各项工作落实，着力增强政府工作的执行力和落实力。坚持依法行政，推进科学民主决策，规范行政执法行为。扎实开展地方政府立法工作，自觉接受人大法律监督、政协民主监督和社会各界监督，认真办理人大代表建议和政协委员提案。落实全面从严治党主体责任，认真履行“一岗双责”，严格落实中央八项规定精神和各项纪律规定，持续加强审计监督，执纪问责力度进一步加大。

　　水库移民、机关事务、外事、侨务、检验检疫、统计、消防、保密、档案、地方志、通信、海事、气象、地震、水文等工作迈出新步伐。老干部、妇女儿童、老龄、残疾人保障等事业取得新进展。工会、共青团、妇联等群团组织在经济社会发展中发挥了重要作用。驻河池中直、区直各单位支持地方发展和自身业绩取得新成效。

　　各位代表，成绩来之不易，奋斗充满艰辛。这些成绩，是习近平新时代中国特色社会主义思想正确指引的结果，是党中央、国务院和自治区党委、政府亲切关怀的结果，是市委团结带领全市各族干部群众努力奋斗的结果，是社会各界倾力相助的结果。在此，我代表市人民政府，向全市各族人民，向人大代表、政协委员，向各民主党派、工商联、无党派人士、人民团体，向驻河池部队指战员、武警官兵、政法干警和民兵预备役人员，向所有关心支持河池发展的各界人士，表示衷心感谢并致以崇高敬意！

　　回顾一年的工作，我们清醒地认识到，全市经济社会发展和政府工作中还存在不少困难和问题：脱贫攻坚任务依然十分艰巨，深度贫困地区产业发展难；高质量发展的基础薄弱，县域经济发展不平衡；交通基础设施仍然落后，招商引资效果不明显，个别重大项目因为政策调整等原因，未能如期开工；政府职能需要进一步转变，干事创业和营商环境需要进一步优化。对这些问题，我们将高度重视，采取得力措施加以解决。

　　二、2019年工作安排

　　今年是新中国成立70周年，是河池决胜脱贫攻坚、全面建成小康社会的关键之年。政府工作的总体要求是：以习近平新时代中国特色社会主义思想为指导，全面贯彻党的十九大和十九届二中、三中全会精神，统筹推进“五位一体”总体布局，协调推进“四个全面”战略布局，坚持稳中求进工作总基调，坚持新发展理念，坚持推动高质量发展，坚持以供给侧结构性改革为主线，落实“三大定位”新使命和“五个扎实”新要求，按照中央、自治区的决策部署和市委“1234”总体工作思路，扎实开展“六大行动” 攻坚年活动，以脱贫攻坚为统领，狠抓产业发展，突出以交通为重点的项目建设，切实抓好稳增长、促改革、调结构、惠民生、防风险各项工作，保持经济持续健康发展和社会大局稳定，为打赢脱贫攻坚战、全面建成小康社会打下决定性基础，为建设壮美广西、共圆复兴梦想贡献力量，以优异成绩迎接中华人民共和国成立70周年。

　　全市经济社会发展的主要预期目标是：地区生产总值增长7%；规模以上工业总产值增长15%、增加值增长8%；固定资产投资增长12%；财政收入增长8%；社会消费品零售总额增长10%；全体居民人均可支配收入增长8%；外贸进出口总额增长6%，其中出口总额增长5%；居民消费价格涨幅控制在3.5%以内。

　　为完成上述目标任务，重点抓好以下工作：

　　（一）全力以赴打好脱贫攻坚战，不断提高精准脱贫质量。深入学习贯彻习近平总书记关于扶贫工作的重要论述，全面实施打赢脱贫攻坚战三年行动，聚焦深度贫困特别是极度贫困地区，精准施策，确保环江、东兰、巴马、凤山4县摘帽，力争罗城摘帽，完成年度贫困村、贫困人口脱贫任务。

　　攻克易地扶贫搬迁“硬骨头”。严格按照“八包”措施，突出抓好搬迁安置点住房及公共服务设施建设，全面完成17.62万建档立卡贫困人口搬迁任务，提前实现“十三五”易地扶贫搬迁目标。落实搬迁后续产业发展帮扶措施，实现每户有1人就业或有1项产业，确保搬迁户搬得出、稳得住、有发展、能致富。

　　继续抓好基础设施建设。加快水、电、路、房、网等基础设施建设。基本完成贫困户、低保户、农村分散供养特困人员和贫困残疾人家庭等对象的危房改造；硬化50户以上自然村道路500公里，对居住相对集中、基础条件较好的200个20户以上自然村实施道路硬化；建设10至19户自然村道路800条1420公里；实施自治区新一轮大石山区农村饮水安全巩固提升大会战，解决30万群众饮水安全问题；抓好新一轮农村电网改造升级；扩大宽带网络覆盖范围，逐步将宽带网络和 4G 网络向有条件的自然村延伸。

　　进一步提升公共服务水平。做好教育扶智工作，资助贫困学生28.6万人次，加大控辍保学力度，不让一名学生因贫失学；因需施策开展“两后生”培训、农民适用技术培训，提高贫困群众自我发展能力。帮扶引导贫困劳动力转移就业，全市农村劳动力转移就业人数稳定在60万人以上。落实医疗救助等优惠政策，贫困群众住院报销比例90%以上，门诊特殊慢性病报销比例80%以上。发挥社保扶贫作用，符合低保条件的贫困户全部纳入低保扶持范围。抓好生态扶贫，开展林业重点项目和生态效益补偿等工作，做好护林员选聘和续聘，增加贫困户工资、经营和补贴收入。

　　拓展对口帮扶广度深度。深化与深圳在产业发展、旅游扶贫、招商引资、转移就业等方面合作，全面完成深圳·河池扶贫协作2018年度项目建设，完成2019年度项目建设任务80%以上。加强中央定点帮扶单位和区直对口帮扶单位联系，争取更多支持。

　　落实精准帮扶责任。深化“5321”结对帮扶制度，做到群众不脱贫，帮扶不脱钩。后盾单位切实履行帮扶职责，督促干部落实帮扶措施。驻村第一书记和工作队员严格按照要求驻村开展工作，遍访贫困户，宣传政策，制定规划，推进实施，确保脱贫各项工作落地生效。帮扶干部要因户精准施策，帮助解决实际问题，为贫困户脱贫致富创造条件，提高群众获得感和满意度。引导贫困群众发扬自力更生精神，激发脱贫内生动力。

　　（二）聚焦乡村振兴战略，促进“十大百万”扶贫产业扩面提质增效。继续把“十大百万”扶贫产业作为农民增收的重要工作来抓，加大产业发展工作力度，落实政策、资金、技术等扶持措施，确保贫困群众有稳定持续的收入来源，助推“三农”工作取得新突破。

　　全面完成扩面任务。各县（区）按照“十大百万”产业年度发展目标，因地制宜，采取切实可行的措施抓好落实，不断扩大产业规模，一定要通过几年的努力，真正打造出3-5个有品牌、有特色、有效益，又能带动群众增收的主导产业。抓好34万亩“万千百”核桃示范基地的管护。全面推广都安“贷牛还牛”模式，整市推进肉牛养殖，打造“河池寿乡牛”品牌，逐步把河池建设成为“中国南方牛都”，全年建设种牛繁育基地11个，发展种牛3万头，养殖肉牛87万头、出栏29万头以上；在全市范围内推广“粮改饲”项目，发展青贮饲料种植55万亩，收贮优质饲草料130万吨以上。充分利用自治区“双千”计划优惠政策加快油茶产业发展，新种油茶18万亩，低产林改造10万亩，每个国有林场创建1个千亩以上高产油茶示范基地。统筹推进其他“十大百万”产业发展，全面完成年度目标任务。

　　发挥示范作用。巩固推进17个示范基地建设，带动现代特色农业示范区增点扩面提质升级，积极创建宜州桑蚕、都安畜牧2个国家级现代农业产业园。新建自治区级农业（林业）核心示范区3个、县级示范区11个、乡级示范园105个、村级示范点524个。

　　拓宽加工销售渠道。培育引进一批规模效益好、市场覆盖面广、带动能力强的龙头企业，抓好农产品精深加工，推动一二三产业融合发展。充分利用深圳帮扶和电商平台，做好线上和线下农产品的宣传和营销服务工作。加大农产品推介展销力度，面向全国、全区开展名特优农产品展销活动。

　　全面推动乡村振兴。坚持规划先行、多规合一，科学编制集住宅建设、道路交通、生态景观、特色产业为一体的乡村振兴建设规划，解决“有新村没新貌”问题。全面实施乡村风貌提升、农村人居环境整治三年行动，年内完成一批实用性村庄规划编制，分类建设一批基本整治型、设施完善型、精品示范型村庄。深入开展“幸福乡村”活动，实施“环境秀美”“生活甜美”“乡村和美”三个专项行动，打造“美丽河池”乡村建设升级版。

　　（三）加快产业转型升级，推动工业高质量发展。积极培育壮大“五大百亿”产业，推动工业结构更优、效益更好。

　　抓好重大工业项目建设。围绕建设生态环保型有色金属产业示范基地以及新技术、新工艺、新动能，谋划一批重大工业项目，补齐产业链条。有色金属、茧丝绸、电力、建材等产业产值同比增长15%、15%、8%、20%以上。突出抓好项目建设，增强工业发展后劲，工业投资增长10%。重点推进24个亿元以上工业项目建设，竣工投产超威鑫锋新型铅蓄电池（二期）、鑫锋环保20万吨废铅酸蓄电池回收、东泰公司40万吨碳酸钙等项目；续建南方公司3万吨锡、12万吨锌，吉朗公司10万吨锌基合金，誉升公司10万吨锌，正华冶炼厂1万吨锑深加工等项目建设；新开工建设博冠公司8.5亿只纸模制品等项目。

　　加快新兴产业发展。积极推动酿酒企业加大技术改造，优化产品结构，提升产品质量，扶持丹泉、天龙泉等企业加强市场开拓工作。引进强优企业整合资源，支持九千万、巴马丽琅等企业做大做强，提高产品知名度和市场占有率。饮用水、酿酒产业产值分别增长20%、8%。推动中国河池——药融园生物医药精细化工项目落户大任产业园，建设河化生物医药产业园，促进生物医药产业集聚发展，实现产值5亿元。

　　抓好工业园区建设。完善园区基础设施，壮大园区规模，五大工业园区实现总产值280亿元。狠抓园区招商引资，每个园区策划亿元以上项目10个，落地5000万元以上项目10个。继续大力推进大任产业园建设，建成园区至拉浪二级公路，开工建设标准厂房，加快推进产业孵化基地项目建设；落实代办服务制，促进企业达产达效，力争园区实现产值30亿元。加快河池·南丹工业园区进出口二级公路建设前期工作，力争开工建设。

　　继续加大服务企业力度。全面落实降成本措施，努力在降低用电价格、减免税费等方面有新成效。建立健全“市长企业接待日活动”以及市、县联系服务企业工作机制，设立工业发展基金，协调金融部门加大对实体经济的扶持力度。扶持龙头企业提高竞争力，引导一批中小企业转型提质，新培育规模以上企业15家。

　　（四）加快服务业发展，为经济发展增添新动能。坚持把发展现代服务业作为高质量发展的潜在动力，依托“世界长寿市”品牌优势，以全域旅游带动，打造经济增长新引擎。

　　加快发展全域旅游。积极推进旅游“双创”，促进旅游业提质增效，全年接待游客增长18%，旅游总消费增长25%。抓好巴马国际旅游区建设，推动单一的景区观光游向融合大健康的康养旅游转变，全面完成巴马国旅区基础设施三年行动计划项目建设，完成投资11亿元；全面启动赐福湖国家度假区建设，重点推进基金小镇、数字小镇、深鹏集团康养综合体、国际生态旅游康养小镇、上海新发展集团赐福养生小镇等项目建设；办好第三届巴马论坛（2019中国·东盟传统医药健康旅游国际论坛）。抓好红水河健康养生之旅建设，推动大化瑶泉、东兰河谷、天峨蓝衣壮等旅游度假区项目取得实质性进展，完成投资5.6亿元。抓好百里龙江旅游带建设，加快推进南丹千家瑶寨·万户瑶乡旅游综合体、丹泉文化旅游创业园、温泉养生度假区（二期），以及金城江珍珠岩养生基地、宜州刘三姐故乡风情旅游区、罗城成龙湖景区、环江木论喀斯特生态旅游区等项目建设，完成投资10亿元；举办南丹·荔波世界白裤瑶乡年街节。充分挖掘红色文化、生态民俗、健康长寿等旅游资源，谋划包装和引进一批项目。加大旅游宣传推介力度，提高河池旅游的知名度和影响力，吸引更多游客到河池康养旅游。

　　积极发展大健康产业。抓好巴马健康养老服务集聚区建设，加快推进深圳·巴马大健康合作特别试验区项目，围绕健康食品、健康服务、健康科技三个核心业态，发展天然饮用水、长寿食品、健康医养、精品体育、会议会展、生物科技、特色医药等七大产业，把巴马建设成为辐射“一带一路”，服务泛珠三角、粤港澳大湾区和东盟乃至全球的大健康产业首善基地。发展森林康养，打造罗城森林康养基地，推进宜州民族花海、天峨红豆杉和东兰玫瑰花海森林特色小镇项目。创建罗城小长安镇等一批广西养生养老小镇。

　　大力发展商贸物流业。启动中心城区商业网点规划修编。开展中心城区肉菜产品、超市、农贸市场追溯体系建设。培育引进区域性大型农产品批发市场、集散中心。推进红星美凯龙商业广场、宜州茧丝绸交易中心、都安畜禽交易中心等商贸综合体及专业市场建设。抓好9个全国电子商务进农村综合示范县项目建设，农村电商覆盖率达60%。强化市场主体培育，稳定进出口企业数量，培育限上商贸服务企业20家。

　　（五）加快重大项目建设，夯实发展后劲。坚持项目谋划和建设两手抓，强化项目支撑引领，以交通的大变化、基础设施的大改善，带动经济社会大发展。

　　强化项目谋划储备。聚焦“一带一路”中新互联互通陆海新通道、粤港澳大湾区、珠江—西江经济带、滇桂黔石漠化片区区域发展与扶贫攻坚规划、左右江革命老区振兴规划等国家和自治区的发展战略，科学谋划一批具有较大投资规模和较强带动作用的项目。全年策划重大项目900个以上，其中5000万元以上项目220个以上，500-5000万元项目680个以上。

　　统筹推进重大项目建设。加快自治区和市级层面统筹推进的 重大项目建设，确保完成投资320亿元以上。狠抓中央预算内投资项目建设，2018年度项目投资完成率90%以上；2019年度项目开工率90%以上，投资完成率60%以上。积极争取和实施左右江革命老区新一轮基础设施大会战项目建设。

　　深入实施交通基础设施建设大会战。全力抓好贵南高铁（河池段）建设；积极推进河池经柳州至贺州、河池至百色高铁项目前期工作。加快贺州至巴马、融水至河池、乐业至百色（天峨段）、南丹至天峨下老4条高速公路建设；力争贵州平塘经天峨、凤山、巴马至南宁，宜州至忻城高速公路具备开工条件，并早日开工建设；加快贵州荔波经环江至德胜高速公路前期工作。继续推进环江至河池一级公路项目。建成贵州瑶山至南丹、南丹吾隘至东兰、天峨至凤山（天峨段）、大化绕城线等二级公路；续建东兰经大化板升至都安大兴、宜州经北牙至龙头、罗城四把至环江、环江英豪至大安、凤山三门海至乐业等一批县乡联网公路。推进龙滩、岩滩升船机和大化船闸按照1000吨级标准建设。落实县（区）主体责任，抓好 “四好农村路”建设。

　　深入实施水利基础设施建设大会战。建成金城江花任、都安大罗、南丹火幕、天峨更旦等4座水库，开工建设罗城板阳东、凤山上林水库，力争南丹天生桥、都安板岭2座中型水库开工，加快金城江水电站、巴马所略水库扩容项目进度，竣工验收凤山乔音水库，加固8座病险水库。加大基本农田水利建设，新增和恢复农田灌溉面积6万亩以上。实施26个河流治理项目，建成抗旱调水工程4个。抓好桂北贫困地区（河池）特色农业高效节水灌溉项目建设，争取批复实施桂西北扶贫治旱—红水河灌区工程项目。

　　抓好电力通信基础设施建设。持续加大电力投资建设力度，加快推进500千伏凤凰变电站，220千伏天峨、东兰、凤山、响泉送变电工程等项目建设，打造安全可靠、绿色高效的河池电网。推进数字河池建设，加快建设新一代信息通信基础设施，完成投资2.35亿元，新建宽带端口5.27万个、基站2387个、网络专线1100条。稳步推进与浪潮集团的合作，加快政务云中心建设。

　　（六）坚持产城融合发展，建设宜居城市。统筹规划、建设、管理，进一步完善功能配套，加快同城发展、产城融合，创造更加宜居的生活环境。

　　推进中心城区建设。编制金宜城市走廊规划，完成金宜一级公路无偿移交并启动项目改造，加快金宜同城化建设步伐。金城江城区建成市体育场、现代职业技术学院实训楼、职教中心实训楼、金城江十小、金城江六中迁建、城西幼儿园、第三幼儿园等项目；开工金城江三中迁建、东江永康小学扩建等项目；提速市本级的人民厂、老虎山两大棚改项目，竣工上任、芝田安置房，启动桥卜、新村安置房建设；完善城区路网，竣工农贸路、塘面路（一期）、桥卜下穿铁路隧道，开工胜利路、桥卜支路，续建辉乐桥、乾霄路。积极推进宜州城区建设，继续抓好中山大道、宜畔路、龙溪路、龙岗路、环城路等城区路网建设。有序推进市行政中心迁移工作。加快宜州新区规划建设，启动园博园、新区路网等项目建设。

　　加强城市精细管理。推动智慧城市建设，推进城市管理人性化、精细化、网格化。严厉打击“两违”，大力开展超限超载、占道经营、道路扬尘、违章停车等专项整治行动，保持城净街美、规范有序。实施生态修复、城市修补“双修”工程，全面加强园林绿化、环卫保洁、物业管理、烟花爆竹管理，倡导绿色生活方式，提高城市宜居水平。

　　推动县域城镇化建设。实施大县城战略，完善县城区基础设施和公共配套建设，推动产业、人口向县城聚集。强化产业引领，每个县引进实施2—3个重大产业项目，培育1—2个特色产业集群。扎实推进县域基础设施能力提升三年行动计划，抓好“路、水、电、网、气、园”等建设，提升县域经济发展支撑能力。统筹推进“东巴凤”一体化发展，规划建设“东巴凤”景区互联及县际互通的交通环路，推动绿色健康产业、生态旅游、城镇建设一体化。推动南丹撤县改市，支持符合条件的乡撤乡改镇。加快各类特色小镇规划建设。

　　（七）打好污染防治攻坚战，加快建设美丽河池。坚定践行习近平生态文明思想，坚持生态严管严治，守护青山绿水，留住蓝天白云，让“河池生态优势金不换”更加深入人心。

　　强化生态保护修复。实施乡村振兴“林业行动”，深入推进森林抚育补贴、森林质量提升、森林景观改造等工程建设，抓好石漠化综合治理，实施百万亩储备林基地建设，完成植树造林25万亩。大力创建国家森林城市，推进城乡绿化一体化。抓好“林长制”试点工作，切实加强生态公益林、天然林的保护。规范自然保护区管理，推进国家湿地公园、森林公园、地质公园等森林景观建设。加大矿山地质环境恢复治理和土地复垦工作力度，抓好地质灾害防治。

　　抓好污染防治。落实“大气十条”，聚焦工业企业排放、城市扬尘、秸秆焚烧等重点污染源，坚持标本兼治，抓住重点地区、重点时段有效施策，市中心城区环境空气质量优良天数92%以上、PM2.5平均浓度低于35微克/立方米。落实“水十条”，深入推行“河长制”，继续加大“清四乱”专项整治，狠抓饮用水安全保障和良好水体保护，主要河流断面水质达标率100%。落实“土十条”，科学编制土壤污染防治规划，坚持“防、控、治”三位一体，加快推进土壤污染综合防治先行区建设，实施污染场地环境风险防范及修复治理、刁江流域农田重金属污染治理等项目建设。

　　落实最严格的生态环境保护制度。巩固中央环保督察组反馈问题整改工作成果，推进环境治理常态化。加大项目环境审查，凡是不符合国家产业政策和环保要求的项目，一律不立项审批；凡是不符合环保标准的企业，一律整顿关停。坚持以“零容忍”的态度依法惩处污染环境、破坏生态的行为。严格落实土地保护制度，严守耕地保护红线。

　　（八）全面深化改革，拓展开放新空间。围绕高质量发展将改革进行到底，深化开放合作，激发市场活力和社会创造力。

　　加大优化营商环境力度。开展“优化营商环境攻坚突破年”活动，推动各项政策和服务落地、落细、落实，提高企业和群众的满意度和获得感。深化“放管服”改革，启用市本级“一站式”新政务服务大厅，政务服务事项做到“应进必进”；扎实推进“一事通办”改革，推动线上“一网通办”，线下“只进一扇门”，现场办理“最多跑一次”，让企业和群众办事像“网购”一样方便。清理规范涉企行政事业性收费，涉及地方规费征收的，可收可不收的，一律不收，有上下限规定的，一律按下限收取。按照“构建亲清新型政商关系”的要求，加大对民营企业的扶持服务力度，积极作为、靠前服务，帮助解决实际困难，激发民营经济活力。

　　抓好重点领域改革。以供给侧结构性改革为主线，落实“三去一降一补”，着力抓好各项改革任务落地。深化国资国企改革，推动国有企业市场化经营，全力支持重点国有企业做大做强做优。深化农业农村改革，完成农村集体产权制度改革工作任务；深化国有林场配套改革，抓好国有林场被侵占林地综合整治工作，推进集体林业综合改革试验区建设；继续推进小型水利工程管理体制改革和农业水价综合改革。抓好第四次全国经济普查、第三次全国土地调查。统筹推进投融资、城市管理、供销合作社、教育、卫生、司法、统计等领域改革。

　　提升开放合作水平。主动融入“一带一路”、粤港澳大湾区、珠江—西江经济带、北部湾经济区建设。加强与贵州黔南州等周边地区联系对接，深化交通基础设施、文化旅游、生态保护等领域合作，实现互联互通、互补互动。加大招商引资力度，开展精准招商，引进一批符合地方产业发展需要、支撑带动能力强的实体经济大项目，提高招商引资项目履约率和落地率，全市招商引资内外资到位同比增长10%。

　　（九）抓好财税金融工作，增强服务经济社会发展能力。坚持打好防范化解重大风险攻坚战，发挥财政政策导向作用，做好金融创新服务和风险防控，保障经济社会持续健康发展。

　　优化财政收支。着力抓好税源转化，拓宽税源广度，夯实财源基础。大力开展城乡建设用地增减挂钩工作，做好土地收储经营，实现资源变资产、生资金。集中财力确保“六大行动”重点项目和各类重点民生支出，为实体经济、项目建设提供资金保障。

　　提升金融服务水平。加大金融服务实体经济、支持脱贫攻坚力度，新增贷款60亿元。利用好贫困县域企业首次公开募股（IPO）优惠政策，引导企业上市挂牌融资。创新绿色金融产品，创建国家林业碳汇扶贫试点。加强政府性融资担保体系建设，扩大融资规模。稳步推进政策性保险，积极发展商业保险。

　　有效防范化解风险。全力化解银行不良资产，严格控制在警戒线内。严厉打击和处置非法集资。加强政府性债务风险评估、预警和监管，防止形成新的隐性债务，牢牢守住不发生系统性区域性金融和债务风险的底线。强化扶贫领域资金监管，加强扶贫小额信贷风险管控，确保资金使用规范、安全、有效。

　　（十）保障和改善民生，增强群众的获得感幸福感安全感。始终树立以人民为中心的发展思想，坚持促发展与惠民生并重，不断满足各族群众日益增长的美好生活需要。

　　发展公平优质教育。推进学前教育，创建多元普惠幼儿园60所，新增园位6000个以上。推进义务教育均衡发展，宜州、罗城、巴马、都安通过国家评估认定。推进普及高中阶段教育，改扩建河池高级中学等7所普通高中，新增普通高中学位3000个。实施中等职业学校布局调整和专业结构优化，改善市职业教育中心等10所学校办学条件，新增中等职业教育学位500个。规范民办教育，抓好特殊教育。配足配齐师资力量，加强教师队伍建设。

　　发展卫生健康事业。实施县级综合医院、妇幼保健院、疾病预防控制中心、乡镇卫生院等63个项目建设，确保年底前全部投入使用。完善分级诊疗制度，80%乡镇卫生院参与医联体建设。传承发展中医药、民族医药事业。提升计划生育和妇幼健康服务水平。

　　提高社会保障能力。全面落实更加积极的就业政策，新增城镇就业1.4万人以上，新增农村劳动力转移就业5万人次。实施全民参保计划，完善城乡居民基本医疗保险、大病保险政策措施。落实职工医疗双保险制度，稳步提高保障水平。提升社会救助、社会福利、残疾人保障服务水平。新开工建设各类保障性住房3900套，基本建成1600套。积极应对人口老龄化，发展居家、社区和互助式养老，用心用情做好老干部和老年人工作，形成尊老敬老的良好社会风尚。

　　推动文化繁荣发展。深入践行社会主义核心价值观，扎实开展新中国成立70周年系列庆祝活动。完善公共文化服务体系，打造河池特色民族文化品牌。繁荣群众文化体育活动，丰富人民精神生活。推进“文化+”行动计划，促进文化与相关产业的融合发展。新建村级公共服务中心100个，完成农家书屋补充更新410个。开展铜鼓文化生态保护实验区（河池）建设。

　　加强和创新社会治理。扎实推进平安河池、法治河池建设，构建多元的化解矛盾纠纷机制，打造共建共治共享的社会治理格局。完善群众诉求表达和社会矛盾排查机制，完成市县乡村四级群众信访服务中心规范化建设。深入推进扫黑除恶专项斗争以及禁毒严打整治等专项行动，依法打击各类违法犯罪行为，持续巩固和提升人民群众安全感、满意度。落实安全生产党政同责、一岗双责，提升应急处置能力，坚决遏制重特大事故发生。落实国土资源执法监管共同责任制，保持国土资源管理良好秩序。强化食品药品安全监管，打造放心消费环境。深化双拥共建，争创全国双拥模范城，抓好退役军人和其他优抚对象的管理服务工作，促进军民融合深度发展。认真贯彻党的民族宗教方针政策，巩固民族团结进步创建成果。

　　三、加强政府自身建设

　　按照习近平总书记提出的信念过硬、政治过硬、责任过硬、能力过硬、作风过硬要求，努力打造人民满意的服务型政府。

　　（一）旗帜鲜明讲政治。坚持用习近平新时代中国特色社会主义思想武装头脑、指导实践、推动工作，树牢“四个意识”，坚定“四个自信”，把准政治方向，严守政治纪律、政治规矩，不忘初心、对党忠诚，坚决维护习近平总书记在党中央的核心、全党的核心地位，坚决维护党中央权威和集中统一领导，坚决贯彻落实习近平总书记重要指示批示和党中央决策部署。

　　（二）深化改革转职能。按照中央、自治区和市委的部署，优化政府机构设置和职能配置，完成机构改革任务，形成职责明确、依法行政的政府治理体系，增强政府公信力和执行力。深化简政放权，优化工作流程，提高政府工作效率和政府服务水平。

　　（三）强化担当抓落实。始终保持锐意进取的精神风貌，把落实作为一种责任、一种担当，力戒形式主义、官僚主义，实干苦干、不务虚功，实实在在办成群众看得见、得实惠的好事实事。完善激励约束、容错纠错机制，旗帜鲜明给积极干事者撑腰鼓劲，对庸政懒政者严肃问责，营造干事创业的良好氛围。

　　（四）依法行政守规范。坚持依法民主决策，构建决策科学、执行坚决、监督有力的权力运行机制。依法接受市人大及其常委会的监督，自觉接受市政协的民主监督，主动接受社会和舆论监督，认真落实市人大及其常委会各项决议决定，办好人大代表建议和政协提案。推进法治政府建设，严格依法行政，把法律约束体现到政府工作全过程。全面推进政务公开，打造阳光透明政府。

　　（五）从严治政树形象。严格贯彻执行中央八项规定，巩固拓展作风建设成果，坚定不移纠“四风”、树新风。加强党风廉政建设，严格落实主体责任，切实履行“一岗双责”。聚焦权力运行风险点，强化审计监督全覆盖，严查扶贫领域腐败和作风问题，保持廉洁高效的政府形象。

　　各位代表！新时代展现美好前景，新使命更加催人奋进。让我们紧密团结在以习近平同志为核心的党中央周围，以习近平新时代中国特色社会主义思想为指导，全面贯彻落实党的十九大精神，在自治区党委、政府和市委的坚强领导下，不忘初心、牢记使命，开拓进取、攻坚克难，推动经济社会持续健康发展，为建设壮美广西、共圆复兴梦想贡献力量！为打赢脱贫攻坚战、同步全面建成小康社会而努力奋斗！
